# Explainable Preoperative Automated Machine Learning Prediction Model for Cardiac Surgery-Associated Acute Kidney Injury

**DOI:** 10.3390/jcm11216264

**Published:** 2022-10-24

**Authors:** Charat Thongprayoon, Pattharawin Pattharanitima, Andrea G. Kattah, Michael A. Mao, Mira T. Keddis, John J. Dillon, Wisit Kaewput, Supawit Tangpanithandee, Pajaree Krisanapan, Fawad Qureshi, Wisit Cheungpasitporn

**Affiliations:** 1 Division of Nephrology and Hypertension, Department of Medicine, Mayo Clinic, Rochester, MN 55905, USA; 2 Department of Internal Medicine, Faculty of Medicine, Thammasat University, Pathum Thani 12120, Thailand; 3 Division of Nephrology and Hypertension, Department of Medicine, Mayo Clinic, Jacksonville, FL 32224, USA; 4 Division of Nephrology and Hypertension, Department of Medicine, Mayo Clinic, Phoenix, AZ 85054, USA; 5 Department of Military and Community Medicine, Phramongkutklao College of Medicine, Bangkok 10400, Thailand; 6 Faculty of Medicine, Chakri Naruebodindra Medical Institute, Ramathibodi Hospital, Mahidol University, Samut Prakan 10540, Thailand

**Keywords:** acute kidney injury, cardiac surgery-associated acute kidney injury, AKI, preoperative, cardiac surgery, machine learning, artificial intelligence, individualized medicine, personalized medicine

## Abstract

Background: We aimed to develop and validate an automated machine learning (autoML) prediction model for cardiac surgery-associated acute kidney injury (CSA-AKI). Methods: Using 69 preoperative variables, we developed several models to predict post-operative AKI in adult patients undergoing cardiac surgery. Models included autoML and non-autoML types, including decision tree (DT), random forest (RF), extreme gradient boosting (XGBoost), and artificial neural network (ANN), as well as a logistic regression prediction model. We then compared model performance using area under the receiver operating characteristic curve (AUROC) and assessed model calibration using Brier score on the independent testing dataset. Results: The incidence of CSA-AKI was 36%. Stacked ensemble autoML had the highest predictive performance among autoML models, and was chosen for comparison with other non-autoML and multivariable logistic regression models. The autoML had the highest AUROC (0.79), followed by RF (0.78), XGBoost (0.77), multivariable logistic regression (0.77), ANN (0.75), and DT (0.64). The autoML had comparable AUROC with RF and outperformed the other models. The autoML was well-calibrated. The Brier score for autoML, RF, DT, XGBoost, ANN, and multivariable logistic regression was 0.18, 0.18, 0.21, 0.19, 0.19, and 0.18, respectively. We applied SHAP and LIME algorithms to our autoML prediction model to extract an explanation of the variables that drive patient-specific predictions of CSA-AKI. Conclusion: We were able to present a preoperative autoML prediction model for CSA-AKI that provided high predictive performance that was comparable to RF and superior to other ML and multivariable logistic regression models. The novel approaches of the proposed explainable preoperative autoML prediction model for CSA-AKI may guide clinicians in advancing individualized medicine plans for patients under cardiac surgery.

## 1. Introduction

Cardiac surgery-associated acute kidney injury (CSA-AKI) is a common and serious complication with incidence ranging from 17% to 49% [1,2,3]. Compared to patients without CSA-AKI, those with CSA-AKI carry increased risks of mortality, prolonged length of hospital stay, and high healthcare costs [4,5,6,7,8]. Previous risk prediction models for CSA-AKI by multivariable logistic regression analysis have been developed with great initiative to help assess perioperative risk of CSA-AKI [9,10,11,12,13,14,15,16,17,18]. However, there are limitations of particular risk scores, such as generalizability (pre-specified type of elective cardiac surgery [9], coronary artery bypass grafting (CABG) [15], or only CKD patients [14]) and the need to include intraoperative factors in the models that are not available for preoperative risk assessment (such as intraoperative inotrope use, intraoperative intra-aortic balloon pump insertion, or cardiopulmonary bypass time [13]). In addition, several risk scores have been developed specifically to predict severe AKI requiring kidney replacement therapy (KRT) after cardiac surgery [10,11,12,16]. However, even milder degrees of CSA-AKI carry increased risks of CKD and progression to end-stage kidney disease (ESKD) and are clinically relevant [3,19,20]. Therefore, there is a need to develop accurate, reliable, and clinically meaningful preoperative risk prediction models for CSA-AKI to assist providers in counseling patients undergoing cardiac surgery. 

Artificial intelligence (AI) and machine learning (ML) have been increasingly applied to individualized medicine [21,22,23,24,25,26], including the prediction of AKI in various settings [27,28,29,30,31,32,33,34,35]. ML algorithms can handle nonlinear, complex, and multidimensional data [36,37], and recent studies have shown high predictive performance from ML algorithms that outperform traditional statistical analyses [38,39]. Recently, automated ML (autoML) has emerged as a growing field to minimize human input and effort on repetitive tasks in ML pipelines, such as optimal algorithm selection and hyperparameter optimization to achieve optimal performance [40], by replacing manual trial-and-error approaches with systematic data-driven decision making [41,42]. In addition, autoML uses automation to efficiently identify the algorithms or models that work best for each dataset and improves accuracy using the ensemble method of algorithms [43]. Thus, autoML has been shown to be very effective, with high predictive performance comparable to human hyperparameter optimization (identification of hyperparameters that returns an optimal model) with a more time-efficient workflow and less human assistance [41,43]. In the present era of utilizing electronic health records (EHRs), where additional data is continuously added and updated, rapid adjustment of the scoring systems in autoML real-world applications is more feasible than traditional ML approaches [40]. Despite the growing research in the field of autoML there has been little work applying autoML to the healthcare field, despite demonstrated need [44].

In this study, we aimed to: (1) develop a preoperative autoML prediction model for CSA-AKI; (2) compare model performance among autoML, various other ML-based prediction models, and traditional statistical (multivariable logistic regression) models in predicting AKI after cardiac surgery in CSA-AKI; and (3) obtain explanations of the features in the ML-based prediction model that drive patient-specific predictions of CSA-AKI. 

## 2. Methods

### 2.1. Patient Population

This was a single-center observational study conducted at a tertiary referral hospital. We studied all consecutive adult patients (≥18 years old) who underwent open-heart surgery at Mayo Clinic Hospital, Rochester, MN, from 1 January 2014 to 31 December 2020. To avoid assessment of multiple outcomes for a single patient, we analyzed only the first heart surgery during the study period for patients with multiple heart surgeries. We excluded (1) patients who had end-stage kidney disease or received any dialysis modalities within 7 days before the surgery, (2) patients who did not have known baseline serum creatinine before surgery, (3) patients who underwent solely right or left ventricular assist device placement, and (4) moribund patients who died during surgery or within 24 h after surgery. The Mayo Clinic Institutional Review Board approved this observational study (IRB number-21-004248) and waived informed consent due to the minimal risk nature of this study. The study was conducted in accordance with the relevant guidelines and regulations.

### 2.2. Data Collection

The primary outcome was post-operative AKI. We defined and staged AKI based solely on the serum creatinine criterion of the Kidney Disease Improving Global Outcomes (KDIGO) foundation [45]; AKI was defined as an increase in serum creatinine of ≥0.3 mg/dL within 48 h after surgery or relative increase of ≥50% from the baseline within 7 days after surgery. We used the most recent outpatient serum creatinine within 1 year prior to the surgery as the baseline value. If the outpatient baseline serum creatinine was not available, we used the lowest in-hospital serum creatinine prior to the surgery as the baseline instead. AKI severity was classified into three stages, as follows: stage 1 was an increase of ≥0.3 mg/dL or an increase to ≥1.5- to 1.9-fold from baseline, stage 2 was an increase to ≥2- to 2.9-fold from baseline, and stage 3 was an increase to >3-fold from baseline, an increase to ≥4.0 mg/dL, or the initiation of renal replacement therapy.

We used our institutional electronic database to abstract cardiac surgery information, patient demographics, comorbidities, echocardiographic findings, vital signs, medications, and laboratory data. Comorbidities were identified according to the Elixhauser Comorbidity index using previously defined ICD-9 and ICD-10 diagnosis codes. As our goal was to develop and assess a prediction model for CSA-AKI based on the available data before cardiac surgery, we only used the preoperative data that were present within 7 days before cardiac surgery for analysis. When multiple values existed, we selected the most recent vital signs or laboratory values prior to cardiac surgery. We excluded laboratory results with more than 10% missing data. Otherwise, we imputed missing data through a multiple imputation approach using Random Forest (RF).

### 2.3. Feature Selection

Spearman’s rank correlation was applied to assess the separate correlation of variables in the dataset and demonstrated no significant correlations (Supplementary Figure S1). Subsequently, a recursive feature elimination (RFE) approach with RF was completed using the Caret R package. The optimal number of variables (69 variables) were identified by the most optimal accuracy and kappa metrics using five times repeated ten-fold cross-validation (Supplementary Figure S2).

### 2.4. Model Development

In order to utilize ML models to predict the risk of AKI after cardiac surgery, we followed TRIPOD (Online Supplementary) to build automated ML and various ML models [46]. Numerical data were normalized to have a standard deviation of 1 and a mean of 0 [47]. 

H_2_O.ai was used to develop autoML models [44]. The H_2_O autoML platform has been validated and provides very stable performance [48]. It includes a number of advanced ML algorithms, including distributed RF (DRF), generalized linear model (GLM), gradient boosting machine (GBM), deep learning (a fully-connected multi-layer neural network), and extremely randomized trees (XRT). In addition, H_2_O-AutoML builds two stacked ensemble models, one using all the trained models and the other using just the best performing model from each algorithm family [49]. Detailed autoML algorithms and hyperparameter optimization processes by H_2_O autoML are provided in the Online Supplementary Materials. 

The overall study cohort was randomized into training (70%), validation (15%), and testing (15%) datasets. The training dataset was used to develop autoML, ML, and traditional multivariable logistic regression analysis models. After model development, autoML models were ranked by evaluation metrics (area under the receiver operating characteristic curve (AUROC) and log loss) on a leaderboard using the validation dataset. The autoML model with highest predictive performance (top-ranked on the leaderboard) was subsequently chosen for comparison with various other ML and traditional multivariable logistic regression analysis models. The testing dataset was blinded to all methods until the final evaluation. As a reference model, we used multivariable logistic regression analysis. We included variables with *p*-value < 0.05 in univariate analysis into the multivariable model and subsequently selected the final multivariable model using a backward stepwise approach with *p*-value < 0.05 as the pre-specified threshold for model retention. 

ML (non-automated) models included decision tree (DT), RF, extreme gradient boosting (XGBoost), and deep learning. We utilized deep learning based on a multi-layer feedforward artificial neural network (ANN) trained with stochastic gradient descent using back-propagation. For DT analysis, the number of terminal nodes was determined considering the scree plot revealing the relationship between the tree size and coefficient of variance. The decision tree was pruned based on cross-validated error results utilizing the complexity parameter associated with the minimal error (Supplementary Figure S3). For the RF model, the number of trees was 500, which yielded the lowest error rate (Supplementary Figure S4), and the mtry value was calculated by the square root of the number of variables [50]. For XGBoost and ANN, we created a hyperparameter tuning grid to identify the best combination of hyperparameters using cross-validation methods (Online Supplementary Data) [51]. 

### 2.5. Model Evaluation and Calibration

The performance of the autoML, ML, and multivariable logistic regression analysis models was assessed with AUROC, accuracy, precision, error rate (ERR), Matthews correlation coefficient (MCC), and F1 score in the testing dataset [52,53,54]. The DeLong test was used to compare AUROCs [55]. Two-sided *p* values less than 0.05 were considered significant. The formula for each measure is provided in the Online Supplementary Data. The Brier score was used to evaluate model calibration [56].

### 2.6. Explanations of the Variables in the autoML-Based Prediction Model That Drive Patient-Specific Predictions of CSA-AKI 

Model-agnostic approaches, including Shapley additive explanations (SHAP) algorithm and Local Interpretable Model-Agnostic Explanations (LIME), were applied to our autoML prediction model in order to extract an explanation of the variables that drive patient-specific predictions to mitigate the issue of black-box predictions [57,58]. 

SHAP is a model-agnostic demonstration of variable importance where the effect of each aspect on a specific prediction is represented through the use of Shapley values [57,58]. The Shapley value indicates how much one singular variable contributes to the difference between the true prediction and the average (mean) prediction in the context of its interaction with other features. In addition, LIME focuses on training local surrogate models to explain individual predictions by building a white-box local surrogate model [58,59]. 

### 2.7. Statistical Analysis

All analyses were performed using R version 4.0.3 (RStudio, Inc., Boston, MA, USA; http://www.rstudio.com/, accessed on 15 July 2021). We used the“h2o” package for autoML and ANN, “rpart” package for DT, “randomForest” and “randomForestExplainer” for RF, “caret” package for RFE variable selection, XGBoost, and grid search, and the “missForest” package for missing data imputation [60].

## 3. Results

### 3.1. Clinical Characteristics

A total of 13,158 cardiac surgery patients were eligible for analysis. The mean age was 65 ± 15 years, and 66% were male. Eighteen percent had coronary bypass graft (CABG), 60% had valve surgery, 19% had CABG and valve surgery, 1% had heart transplant, and 2% had pericardiectomy. The mean baseline creatinine was 1.1 ± 0.7 mg/dL and the estimated glomerular filtration rate was 69± mL/min/1.73 m^2^ (Table 1). Thirty-six percent (*n* = 4745) developed CSA-AKI, with 30% in stage 1, 3% in stage 2, and 3% in stage 3. Two percent (*n* = 284) required postoperative renal replacement therapy.

Of these eligible cardiac surgery patients, 9244, 1967, and 1947 were randomly included in the training, validation, and testing dataset, respectively. Table 1 shows the clinical characteristics of patients in the training, validation, and testing datasets. Clinical characteristics among the training, validation, and testing datasets were mostly comparable. The incidence of CSA-AKI was similar among the three datasets (36% in training vs. 36% in validation vs. 35% in testing; *p* = 0.73).

### 3.2. AutoML Prediction Models for CSA-AKI

AutoML models for CSA-AKI were developed in the training dataset and were ranked by AUROC and log loss on the leaderboard using the validation dataset (Supplementary Table S1). 

Table 2 demonstrates the top 20 autoML models for CSA-AKI. The top autoML (Stacked ensemble model ID: StackedEnsemble_AllModels_3_AutoML_1_20211031_170047) shows the highest predictive performance on the leaderboard (AUROC = 0.78), and thus was subsequently chosen for comparison with other various ML and traditional multivariable logistic regression analysis models.

### 3.3. Traditional Logistic Regression Prediction Model for CSA-AKI

In the final multivariable logistic regression, the predictors for CSA-AKI included age, sex, race, cardiac surgery type, history of cardiac arrhythmia, peripheral vascular disease, hypertension with and without complications, liver disease, coagulopathy, obesity, right ventricular systolic pressure, systolic blood pressure, the use of aspirin, beta-blockers, anti-arrhythmic medications, benzodiazepine, vasopressor/inotropes, insulin, serum sodium, albumin, hemoglobin, and eGFR. (Supplementary Table S2).

### 3.4. Model Comparison among the Different Models

**Figure 1:**
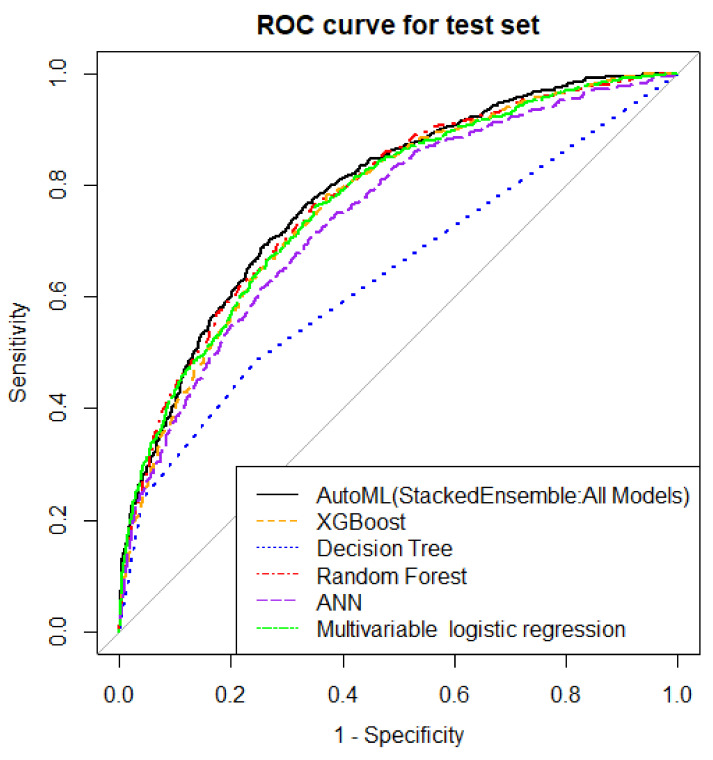
Comparison of AUROC among autoML model, different ML models, and logistic regression model. AUROC, area under the receiver operating characteristic curve; ML, machine learning.

**Figure 2:**
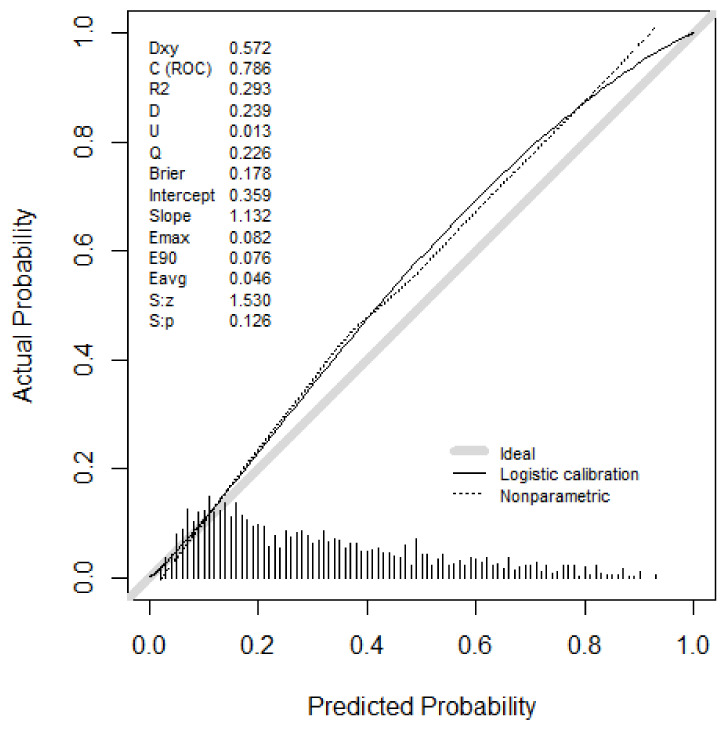
Calibration plot autoML. Brier: Brier score; C (ROC), AUC for discrimination; D, discrimination index; Dxy, Somer’s rank correlation; Emax/E90/Eavg: Maximum/90th quantile, average absolute difference in predicted and smoothed calibrated probabilities; Q, quality index; R2: Nagelkerke-Cox-Snell-Maddala-Magee R-squared index; S:z/S:p the z and two-sided *p*-value of the Spiegelhalter test for calibration accuracy; U, unreliability index.

The ERRs, accuracy, precision, MCC, F1 score, and AUROCs of the top autoML, all ML models, and the multivariable logistic regression model for CSA-AKI prediction in the test dataset are shown in Table 3 and Figure 1. DT showed the highest ERR (29.6%) and the lowest accuracy (0.70), MCC score (0.30), F1 score (0.36), and AUROC (0.64, 95% confidence interval (CI): 0.62–0.66). AUROCs were comparable among autoML (0.79 (95%CI: 0.77–0.81)) and RF model 0.78 (95%CI: 0.76–0.80), *p* = 0.07. The autoML model outperformed DT (AUROC 0.64 (95%CI: 0.62–0.66), *p* < 0.01), XGBoost (AUROC 0.77 (95%CI: 0.75–0.79), *p* < 0.01), ANN (AUROC 0.75 (95%CI: 0.72–0.77), *p* < 0.01), and multivariable logistic regression model (AUROC 0.77(95%CI: 0.75–0.79) *p* = 0.01). The autoML model was well-calibrated (Figure 2). The Brier scores for autoML, RF, DT, XGBoost, ANN, and multivariable logistic regression were 0.18, 0.18, 0.21, 0.19, 0.19, and 0.18, respectively.

### 3.5. Explanations of the Variables in the autoML-Based Prediction Model That Drive Patient-Specific Predictions of CSA-AKI

**Figure 3:**
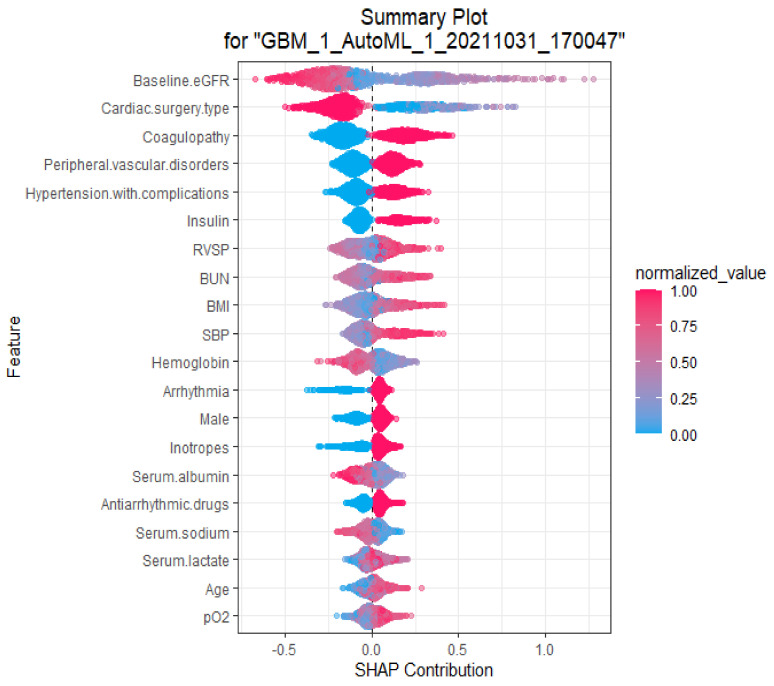
SHAP summary plot of the top 20 features of the GBM autoML (model ID: GBM_1_AutoML_1_20211031_170047), which is one of the key models in the component of our top autoML model. The higher the SHAP value of a feature, the higher the probability of CSA-AKI. Abbreviations: BMI, body mass index; BUN, blood urea nitrogen; eGFR, estimated glomerular filtration rate; pO2, partial pressure of oxygen; RVSP, right ventricular systolic pressure; SBP, systolic blood pressure.

To identify the features that influenced the autoML prediction model the most, we applied the SHAP algorithm to our autoML prediction model in order to extract an explanation of the variables that drive patient-specific predictions for CSA-AKI. As the SHAP algorithm could be utilized for the ensemble model, it was applied to GBM_1_AutoML_1_20211031_170047 (rank number 7 on the leaderboard Table 2), which was one of the key models in the component of our top autoML model (Stacked ensemble model ID: StackedEnsemble_AllModels_3_AutoML_1_20211031_170047). The SHAP summary plot of GBM_1_AutoML_1_20211031_170047 model and the top 20 features of the prediction model are shown in Figure 3. This plot depicts how high and low the feature values were in relation to the SHAP values in the testing dataset. According to the prediction model, the higher the SHAP value of a feature, the higher probability of CSA-AKI occurring. Top 3 features that influenced predictions of CSA-AKI included baseline eGFR, cardiac surgery type, and coagulopathy, respectively. 

**Figure 4:**
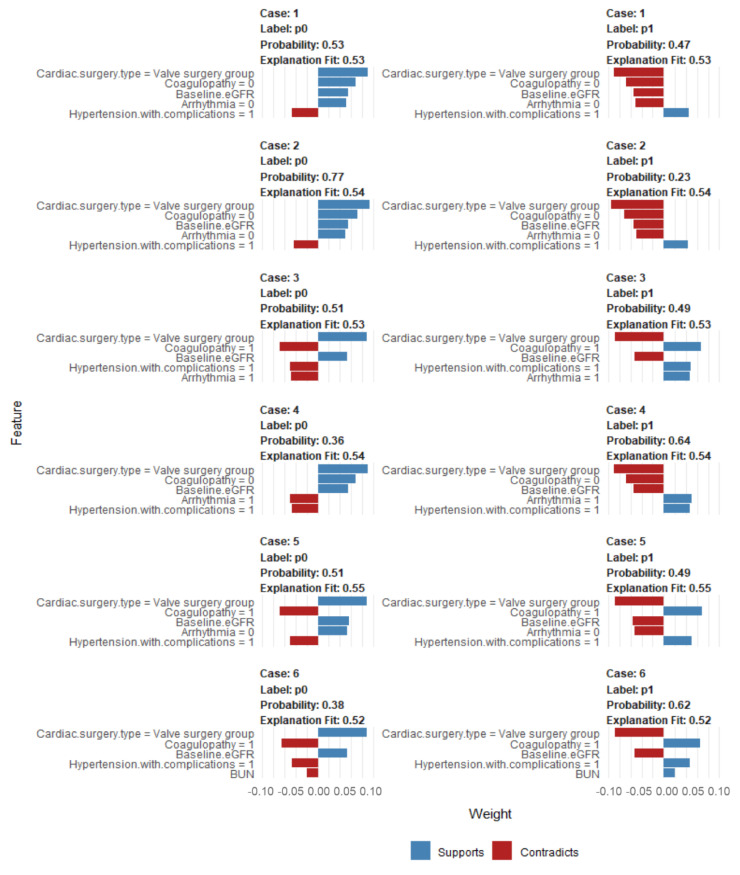
Local interpretable model explainer (LIME) of top autoML (model ID: StackedEnsemble_AllModels_3_AutoML_1_20211031_170047) for six individual cases (case# 1 to 6) from the testing dataset. Label “1” means prediction of CSA-AKI and label “0” means prediction of no CSA-AKI. Probability shows the probability of the observation belong to the label “1” or “0”. The five most important features that best explain the linear model in that observation’s local region are demonstrated along with whether the features influence an increase in the probability (blue bar/supports or a decrease in the probability (red bar/contradicts). The x-axis demonstrated how much each feature added or subtracted to the final probability value for the patient. Abbreviations: BUN, blood urea nitrogen; eGFR, estimated glomerular filtration rate.

Additionally, we applied LIME into autoML model to illustrate the impact of key variables at the individual level (Figure 4). For each patient and individual risk assessment of CSA-AKI a LIME plot was generated depicting the top five variables that support (increase the risk of CSA-AKI) or contradict (decrease the risk of CSA-AKI) the prediction of CSA-AKI for each patient.

## 4. Discussion

Significant efforts have been invested in the development of predictive risk models of CSA-AKI. Traditional statistical models such as logistic regression analysis have been previously utilized to construct such prognostication tools [9,10,11,12,13,14,15,16,17]. In recent years, ML predictive algorithms have emerged as a method to handle high-dimensional, unstructured, and complex structured data including hospitalized patient with AKI [27,28,29,30,31]. While autoML has been shown to be very effective, with high predictive performance comparable to human hyperparameter optimization and with higher time-efficient workflow when compared to non-automated ML [41,43], autoML has never been utilized in the development of AKI prediction models. In this study, we successfully developed preoperative autoML prediction models for CSA-AKI and compared the predictive performances of autoML models with unautomated ML, and conventional multivariable logistic regression models.

Previous traditional risk prediction models using multivariable logistic regression for CSA-AKI have been developed [9,10,11,12,13,14,15,16,17,18], including those with risk scores that included only subgroups of patients undergoing cardiac surgery, such as elective cardiac surgery [9], CABG [15], or only patients with CKD [14]. While the inclusion of intraoperative variables in the risk scores helps to improve predictive performances [13], the utilization of these models is limited in real clinical practice of preoperative risk assessment of CSA-AKI. In addition, several risk scores have been developed specifically to predict severe AKI requiring KRT after cardiac surgery [10,11,12,16]. Considering that CSA-AKI, even with milder severity of AKI, involves increased risks of CKD and ESKD [3,19,20], in the current era of individualized medicine and advanced EHR the development of preoperative ML risk prediction models for CSA-AKI can be clinically meaningful to assist providers in the counseling of each individual patient prior to cardiac surgery. Recently, there has been increasing interest in the utilization of supervised non-automated ML algorisms to predict the risk of CSA-AKI [32,33,61,62]. While these ML models provide excellent discrimination of cases with CSA-AKI [32,33,61] and higher predictive performances than traditional multivariable logistic regression analyses, these non-automated ML predictive models for CSA-AKI include intraoperative data in order to achieve high predictive performance [32,33,61]. Thus, the utilizations of these ML models for preoperative risk assessment are limited. 

Our study solely used preoperative data in the development of CSA-AKI prediction models. Additionally, for the first time we utilized the autoML approach in the development of preoperative prediction models for CS-AKI. Furthermore, we demonstrated that the top autoML from the leader board (stacked ensemble model ID: StackedEnsemble_AllModels_3_AutoML_1_20211031_170047) achieved optimal predictive performance, as demonstrated in non-automated RF, and outperformed the DT, XGBoost, ANN, and multivariable logistic regression model. In addition to high predictive performance, the autoML approach requires less human assistance and reduces human biases in optimal algorithm selection and hyperparameter optimization of model development [43]. With the rapid changes in novel treatment patterns, demographics, and patient populations, data shifts have been increasingly recognized and have significantly affected predictive performance over time [63,64]. The rapid adjustment of autoML predictive performance with new data is more feasible than non-automated ML models [40], and can improve time-efficient workflow in the model maintenance phase. 

One issue that has received considerable visibility and has often been cited as a limitation on the use of ML and autoML in clinical applications is a lack of transparency and interpretability in ML-derived recommendations [57,58]. When provided two models of equal performance, one a black box model and one an interpretable model, most users opt for the interpretable model [65]. Gaining user trust has frequently been referenced as one reason for interpretability [66]. In this study, to obtain explanations of the variables that drive patient-specific predictions of CSA-AKI, we applied model-agnostic approaches to our autoML prediction models using the SHAP and LIME algorithms [57,58]. While SHAP cannot be used with our top autoML model, as it is an ensemble model that combines several base models in order to produce one optimal predictive model, we applied the SHAP algorithm to explain the top 20 variables that played the most important roles in predicting of CSA-AKI in GBM autoML (model ID: GBM_1_AutoML_1_20211031_170047), which is one of the key models in the component of our top autoML model. The LIME algorithm can be utilized for ensemble models, and thus we successfully applied it to our top autoML prediction model. Through the adoption of the LIME approach, we were able to explain variables driving patient-specific predictions of CSA-AKI for each individual patient and reduce the black box concern of our preoperative autoML prediction model for CSA-AKI.

There are several limitations of our study. First, our study cohort represents a majority Caucasian population, and thus the autoML prediction model may need further adjustment with more data including other patient populations. Second, our autoML included only preoperative data in order to make it applicable in real clinical practice for preoperative assessment. While incorporation of intraoperative factors such as operative time and cardiopulmonary bypass time may additionally improve model predictive performance of CSA-AKI, and may be beneficial for interventional research during or after cardiac surgery, this is not the main focus of our current study. Lastly, a future validation study and external validation studies of preoperative autoML prediction models for CSA-AKI are needed. 

## 5. Conclusions

In conclusion, we presented a preoperative autoML prediction model for CSA-AKI (available online as a shiny app at https://wisitc.shinyapps.io/autoML-CSA-AKI/, created on 21 July 2022)) that provided high predictive performance comparable to non-automated ML approaches, and superior to the multivariable logistic regression model. In addition, we demonstrated the explainability of our preoperative autoML prediction model for CSA-AKI. These novel approaches involving an explainable preoperative autoML prediction model for CSA-AKI may guide clinicians in advancing individualized medicine plans for patients under cardiac surgery. 

## Figures and Tables

**Table 1:** Patient characteristics in the datasets.

Characteristics	All	Training	Validation	Testing	*p*-Value
	(*n* = 13,158)	(*n* = 9244)	(*n* = 1967)	(*n* = 1947)	
Age (years)	65 ± 15	65 ± 15	65 ± 15	65 ± 15	0.67
Male sex	8642 (66)	6066 (66)	1335 (68)	1241 (64)	0.02
Race					0.49
White	12,460 (95)	8753 (95)	1857 (94)	1850 (95)	
Black	164 (1)	112 (1)	23 (1)	29 (2)	
Asian	213 (2)	155 (2)	29 (2)	29 (1)	
Other	321 (2)	224 (2)	58 (3)	39 (2)	
Body mass index (kg/m^2^)	29.7 ± 6.5	29.7 ± 6.5	29.6 ± 6.3	29.9 ± 6.8	0.31
Admission type					0.72
Elective	11,020 (84)	7728 (83)	1659 (84)	1633 (84)	
Urgent	1396 (11)	988 (11)	195 (10)	213 (11)	
Emergent	742 (5)	528 (6)	113 (6)	101 (5)	
Cardiac surgery type					0.11
CABG	2308 (18)	1592 (17)	357 (18)	359 (18)	
Valve surgery	7920 (60)	5575 (60)	1145 (58)	1200 (62)	
CABG + valve surgery	2503 (19)	1765 (19)	408 (21)	330 (17)	
Heart transplant	109 (1)	79 (1)	16 (1)	14 (1)	
Pericardiectomy	318 (2)	233 (3)	41 (2)	44 (2)	
Comorbidity					
Congestive heart failure	9658 (73)	6804 (74)	1429 (73)	1425 (73)	0.67
Arrhythmia	10,370 (79)	7279 (79)	1535 (78)	1556 (80)	0.34
Valvular disease	11,144 (85)	7854 (85)	1649 (84)	1641 (84)	0.39
Peripheral vascular disease	6281 (48)	4456 (48)	903 (46)	922 (47)	0.17
Hypertension; uncomplicated	2643 (20)	1857 (20)	418 (21)	368 (19)	0.19
Hypertension; complicated	5334 (40)	3806 (41)	740 (38)	788 (40)	0.01
Paralysis	182 (1)	130 (1)	24 (1)	28 (1)	0.79
Neurological disorders	390 (3)	281 (3)	65 (3)	44 (2)	0.11
COPD	3049 (23)	2139 (23)	443 (22)	467 (24)	0.55
Diabetes; no complications	2573 (20)	1807 (19)	392 (20)	374 (19)	0.85
Diabetes; complications	2011 (15)	1412 (15)	292 (15)	307 (16)	0.72
Hypothyroidism	2025 (15)	1417 (15)	294 (15)	314 (16)	0.57
Liver disease	663 (5)	482 (5)	87 (4)	94 (5)	0.31
Peptic ulcer disease	77 (1)	51 (1)	15 (1)	11 (1)	0.53
Lymphoma	132 (1)	89 (1)	19 (1)	24 (1)	0.55
Solid cancer	285 (2)	202 (2)	43 (2)	40 (2)	0.93
Connective tissue disease	639 (5)	448 (5)	78 (4)	113 (6)	0.03
Coagulopathy	5651 (43)	4035 (44)	849 (43)	767 (39)	0.003
Obesity	3713 (28)	2585 (28)	559 (28)	569 (29)	0.52
Weight loss	263 (2)	167 (2)	50 (2)	46 (2)	0.04
Blood loss anemia	152 (1)	112 (1)	20 (1)	20 (1)	0.65
Anemia	600 (5)	415 (4)	95 (5)	90 (5)	0.8
Drug abuse	200 (1)	146 (2)	26 (1)	28 (1)	0.66
Psychosis	57 (0)	39 (0)	12 (1)	6 (0)	0.34
Depression	1683 (13)	1175 (13)	258 (13)	250 (13)	0.88
Echo finding					
LVEF	57.8 ± 9.4	57.8 ± 9.5	57.8 ± 9.5	57.9 ± 9.3	0.85
RVSP	38.5 ± 10.9	38.5 ± 11.0	38.3 ± 10.9	38.4 ± 10.7	0.54
Systolic blood pressure (mmHg)	130.4 ± 17.4	130.3 ± 17.6	130.0 ± 16.9	130.9 ± 17.3	0.14
Diastolic blood pressure (mmHg)	72.8 ± 11.8	72.8 ± 11.8	72.9 ± 11.7	72.8 ± 11.7	0.9
IABP use	242 (2)	173 (2)	33 (2)	36 (2)	0.84
Medications					
Aspirin	2257 (17)	1565 (17)	351 (18)	341 (17)	0.56
Beta-blockers	2739 (21)	1914 (21)	436 (22)	389 (20)	0.22
Digoxin	180 (1)	123 (1)	27 (1)	30 (1)	0.77
Anti-anginal medications	1666 (13)	1163 (13)	254 (13)	249 (13)	0.91
Anti-arrhythmic medications	7296 (55)	5154 (56)	1075 (55)	1067 (55)	0.55
Statins	1843 (14)	1282 (14)	293 (15)	268 (14)	0.46
ACEIs	695 (5)	499 (5)	117 (6)	79 (4)	0.02
ARBs	300 (2)	212 (2)	44 (2)	44 (2)	0.99
NSAIDs	868 (7)	626 (7)	114 (6)	128 (7)	0.28
Benzodiazepine	7990 (61)	5658 (61)	1172 (60)	1160 (60)	0.22
Vancomycin	11 (0)	8 (0)	2 (0)	1 (0)	0.85
Contrast	730 (5)	518 (6)	113 (6)	99 (5)	0.61
Diuretics	1569 (12)	1105 (12)	230 (12)	234 (12)	0.94
Calcium channel blockers	886 (7)	620 (7)	136 (7)	130 (7)	0.94
Vasopressors/inotropes	9232 (70)	6488 (70)	1401 (71)	1343 (69)	0.31
Insulin	3899 (30)	2756 (30)	580 (29)	563 (29)	0.21
Laboratory data					
Sodium (mEq/L)	137.6 ± 3.7	137.6 ± 3.7	137.4 ± 3.7	137.7 ± 3.7	0.04
Potassium (mEq/L)	4.2 ± 0.6	4.3 ± 0.6	4.3 ± 0.6	4.3 ± 0.6	0.96
Chloride (mEq/L)	101.7 ± 3.0	101.7 ± 3.0	101.7 ± 3.0	101.9 ± 3.0	0.18
Bicarbonate (mEq/L)	25.3 ± 2.5	25.3 ± 2.5	25.3 ± 2.4	25.2 ± 2.5	0.5
BUN (mg/dL)	20.2 ± 10.0	20.2 ± 10.0	19.8 ± 9.3	20.5 ± 10.6	0.09
Ionized calcium (mmol/L)	4.4 ± 0.4	4.4 ± 0.4	4.4 ± 0.4	4.4 ± 0.4	0.96
Glucose (mg/dL)	117.8 ± 32.5	117.5 ± 32.4	118.6 ± 33.1	118.3 ± 32.5	0.32
Albumin (g/dL)	4.1 ± 0.3	4.1 ± 0.4	4.1 ± 0.4	4.1 ± 0.4	0.82
pH	7.4 ± 0.1	7.4 ± 0.1	7.4 ± 0.1	7.4 ± 0.1	0.84
pO2 (mmHg)	275.2 ± 98.4	275.2 ± 98.2	274.3 ± 98.2	276.4 ± 99.4	0.8
hemoglobin (g/dL)	11.5 ± 2.0	11.5 ± 2.0	11.5 ± 2.0	11.5 ± 2.0	0.9
WBC (109 cells/L)	7.1 ± 3.4	7.1 ± 3.4	7.1 ± 2.7	7.2 ± 3.7	0.34
Platelet (109 cells/L)	214.0 ± 70.2	213.7 ± 70.7	212.5 ± 68.1	216.6 ± 70.1	0.16
INR	1.2 ± 0.3	1.2 ± 0.3	1.2 ± 0.3	1.2 ± 0.3	0.43
Lactate (mmol/L)	1.2 ± 0.6	1.2 ± 0.6	1.2 ± 0.6	1.2 ± 0.7	0.9
eGFR (mL/min/1.73 m^2^)	69.2 ± 21.2	69.1 ± 21.3	69.8 ± 20.8	68.7 ± 21.2	0.24
positive blood culture	59 (0)	46 (0)	9 (0)	4 (0)	0.21
Outcome					
Acute Kidney Injury	4745 (36)	3342 (36)	716 (36)	687 (35)	0.73

Abbreviations: ACEI, angiotensin-converting enzyme inhibitors; ARBs, Angiotensin II receptor blockers; BUN, blood urea nitrogen; CABG, coronary artery bypass graft surgery; COPD, chronic obstructive pulmonary disease; eGFR, estimated glomerular filtration rate; IABP, intra-aortic balloon pump; INR, international normalized ratio; LVEF, left ventricular ejection fraction; NSAIDs, non-steroidal anti-inflammatory drugs; pH, potential of hydrogen; pO2, partial pressure of oxygen; RVSP, right ventricular systolic pressure; WBC, white blood cell.

**Table 2:** Leaderboard of top 20 autoML models for CSA-AKI ranked by evaluation metrics using validation dataset.

Rank	Model ID	AUROC	Log loss
1	StackedEnsemble_AllModels_3_AutoML_1_20211031_170047	0.777477459373283	0.546459347839992
2	StackedEnsemble_AllModels_2_AutoML_1_20211031_170047	0.773762554202448	0.541472780910445
3	StackedEnsemble_AllModels_1_AutoML_1_20211031_170047	0.773350035055754	0.541923951699646
4	StackedEnsemble_BestOfFamily_1_AutoML_1_20211031_170047	0.773241741802089	0.541880114043628
5	StackedEnsemble_BestOfFamily_3_AutoML_1_20211031_170047	0.772737675781163	0.543015006080206
6	StackedEnsemble_BestOfFamily_2_AutoML_1_20211031_170047	0.772442939503146	0.542787093883418
7	GBM_1_AutoML_1_20211031_170047	0.771870771539193	0.545029939918007
8	GBM_grid_1_AutoML_1_20211031_170047_model_2	0.77171223914723	0.544501614697186
9	GBM_grid_1_AutoML_1_20211031_170047_model_11	0.770116309187287	0.546966245682808
10	GBM_grid_1_AutoML_1_20211031_170047_model_16	0.769074126173921	0.545687661410384
11	GBM_grid_1_AutoML_1_20211031_170047_model_6	0.768387524617178	0.546875946078973
12	GBM_5_AutoML_1_20211031_170047	0.767743347221664	0.547846265522666
13	GBM_grid_1_AutoML_1_20211031_170047_model_14	0.765551804366563	0.55048346881313
14	GBM_grid_1_AutoML_1_20211031_170047_model_7	0.764637452049534	0.551072950563168
15	GBM_3_AutoML_1_20211031_170047	0.763708027991015	0.549131275569399
16	GBM_grid_1_AutoML_1_20211031_170047_model_1	0.763258108596921	0.549864223764978
17	GBM_2_AutoML_1_20211031_170047	0.761695113183196	0.553063273816373
18	GBM_grid_1_AutoML_1_20211031_170047_model_10	0.75964423991533	0.553470882528734
19	GBM_grid_1_AutoML_1_20211031_170047_model_9	0.759394718861782	0.554178650562614
20	GBM_grid_1_AutoML_1_20211031_170047_model_12	0.757099906666845	0.555638148301273

Abbreviation: AUROC, area under the receiver operating characteristic curve; autoML, automated machine learning; CSA-AKI, cardiac surgery-associated acute kidney injury; GBM, gradient boosting machine.

**Table 3:** Comparison of evaluation metrics and calibration among the different models.

Model	Error Rate of Test Data Set	Accuracy	Precision	MCC	F1 Score	AUROC in the Test Set	Brier Score
AutoML (StackedEnsemble_AllModels_3_AutoML_1_20211031_170047)	27.6%	0.72	0.71	0.35	0.49	0.79 (0.77–0.81)	0.18
Random forest model	26.4%	0.74	0.71	0.39	0.54	0.78 (0.76–0.80)	0.18
Decision tree	29.6%	0.70	0.75	0.30	0.36	0.64 (0.62–0.66)	0.21
XGBoost	27.8%	0.72	0.65	0.36	0.53	0.77 (0.75–0.79)	0.19
ANN	29.1%	0.71	0.78	0.32	0.37	0.75 (0.72–0.77)	0.19
Multivariable logistic regression	27.0%	0.73	0.67	0.38	0.54	0.77 (0.75–0.79)	0.18

Abbreviation: ANN, artificial neural network; AUROC, area under the receiver operating characteristic curve; MCC: worst value −1 and best value +1. F1 score, accuracy, and precision: worst value 0 and best value 1. The Brier score is a combined measure of discrimination and calibration that ranges between 0 and 1, where the best score is 0 and the worst is 1.

## Data Availability

Data are available upon reasonable request to the corresponding author.

## Supplementary Materials

The following supporting information can be downloaded at: https://www.mdpi.com/article/10.3390/jcm11216264/s1, Figure S1: Correlation of variables in the dataset; Figure S2: The optimal number of variables (69 variables) were identified by the most optimal (A) accuracy and (B) kappa metrics using 5 times repeated 10-fold cross validation; Figure S3: Pruned decision tree associated with the minimal error; Figure S4: Number of trees of RF model which yielded the lowest error rate; Figure S5: Simple decision tree model showing the classification of patients who had CSA-AKI (1) and did not (0) have CSA-AKI. Supplementary Table S1 Leaderboard of top 45 autoML models for CSA-AKI ranked by evaluation metrics using validation dataset. Supplementary Table S2. Development of multivariable logistic regression model to predict acute kidney injury after cardiac surgery using stepwise variable selection in the training dataset. TRIPOD Checklist: Prediction Model Development
