# Supplementary material for: Explainable Preoperative Automated Machine Learning Prediction Model for Cardiac Surgery-Associated Acute Kidney Injury
Source: J Clin Med. 2022 Oct 24;11(21):6264. doi: 10.3390/jcm11216264 (PMC9656700; doi:10.3390/jcm11216264)
Supplement: Supplementary file 1 [file jcm-11-06264-s001.zip › jcm-1936903-supplementary.pdf]

Supplementary Figure S1: Spearman's rank correlation demonstrated no significant correlations

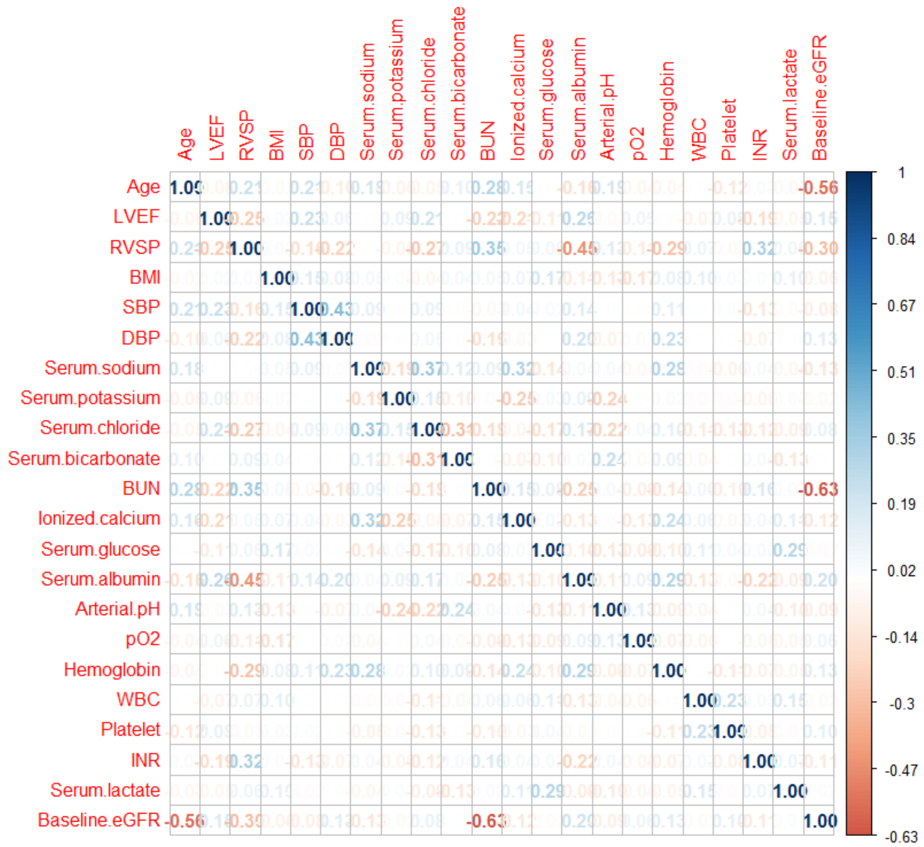

Supplementary Figure S2: The optimal number of variables (69 variables) were identified by the most optimal A) accuracy and B) kappa metrics using 5 times repeated 10-fold cross validation.

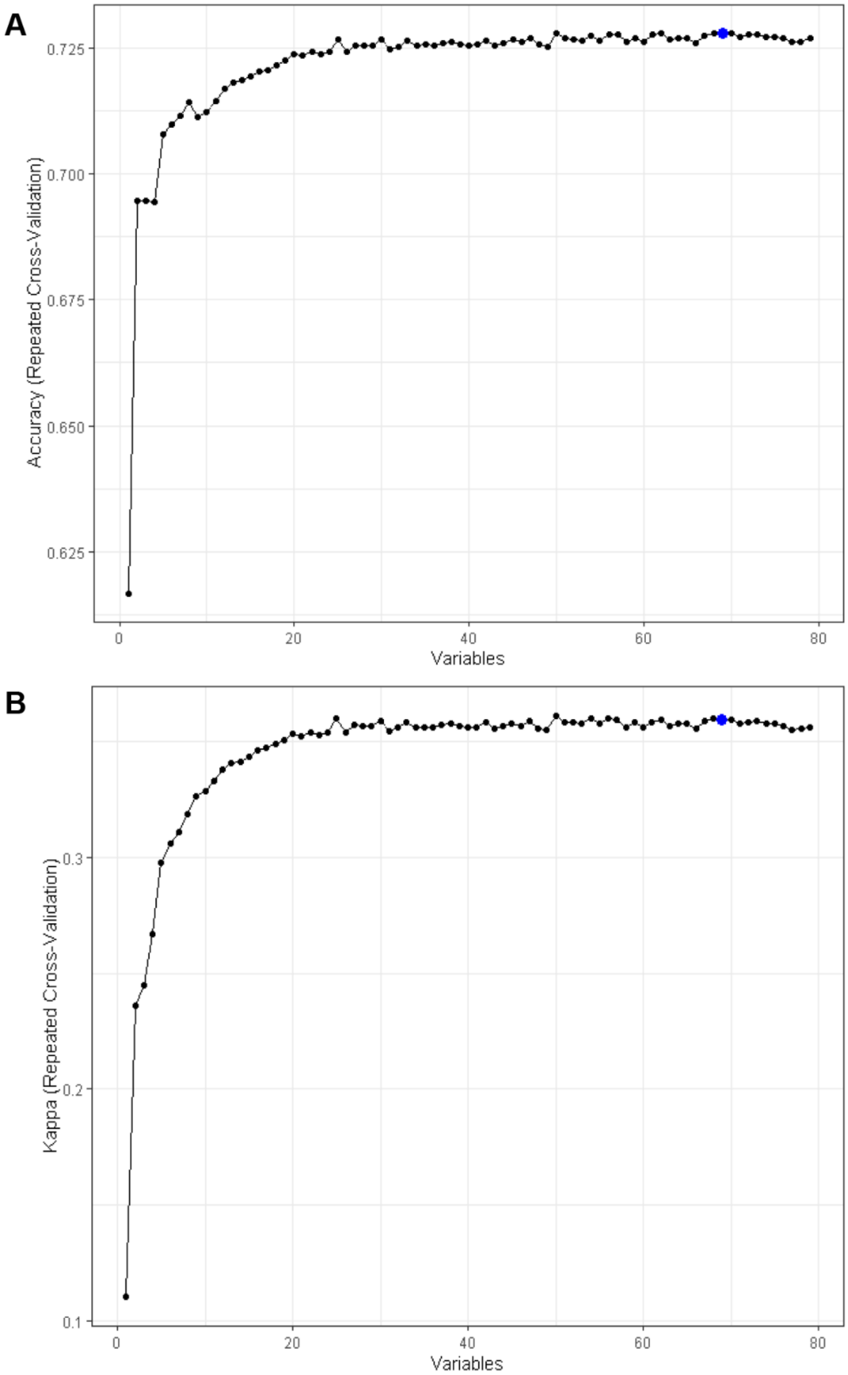

Supplementary Figure S3: Pruned decision tree associated with the minimal error

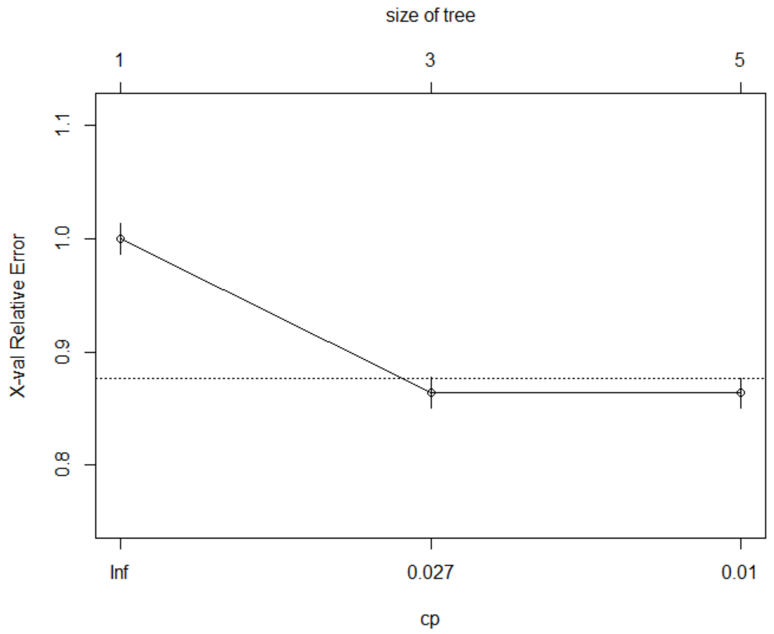

Supplementary Figure S4: Number of trees of RF model which yielded the lowest error rate

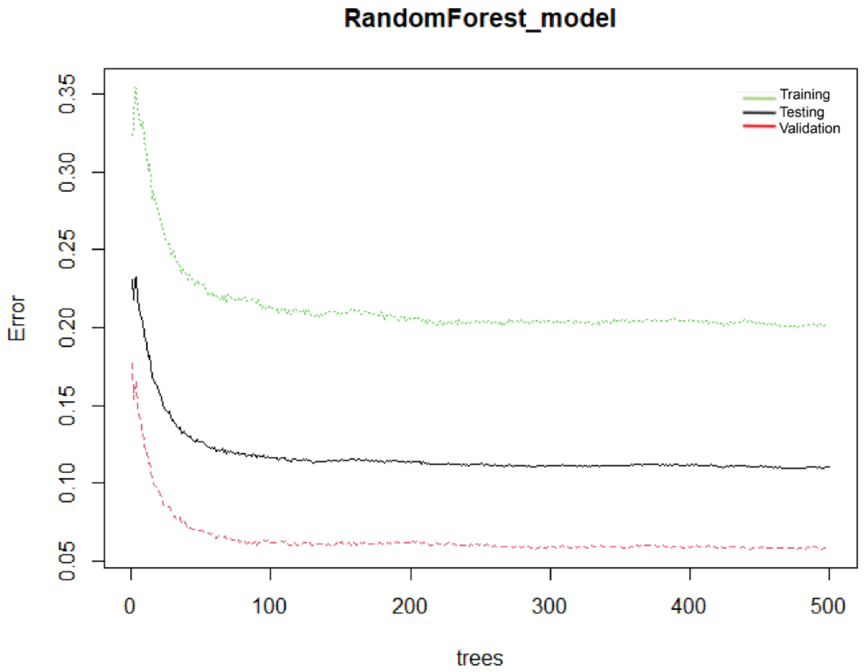

Supplementary Figure S5: Simple decision tree model showing the classification of patients who had CSA-AKI (1) and did not (0) have CSA-AKI. The numbers with two decimals in each cell means the probability of mortality in each classification tree. The blue or green color becomes dense when it is more likely to die or not. The % number in the boxes denotes the percentage of patients with each discriminating variable from CART (Classification and Regression Tree) analysis.

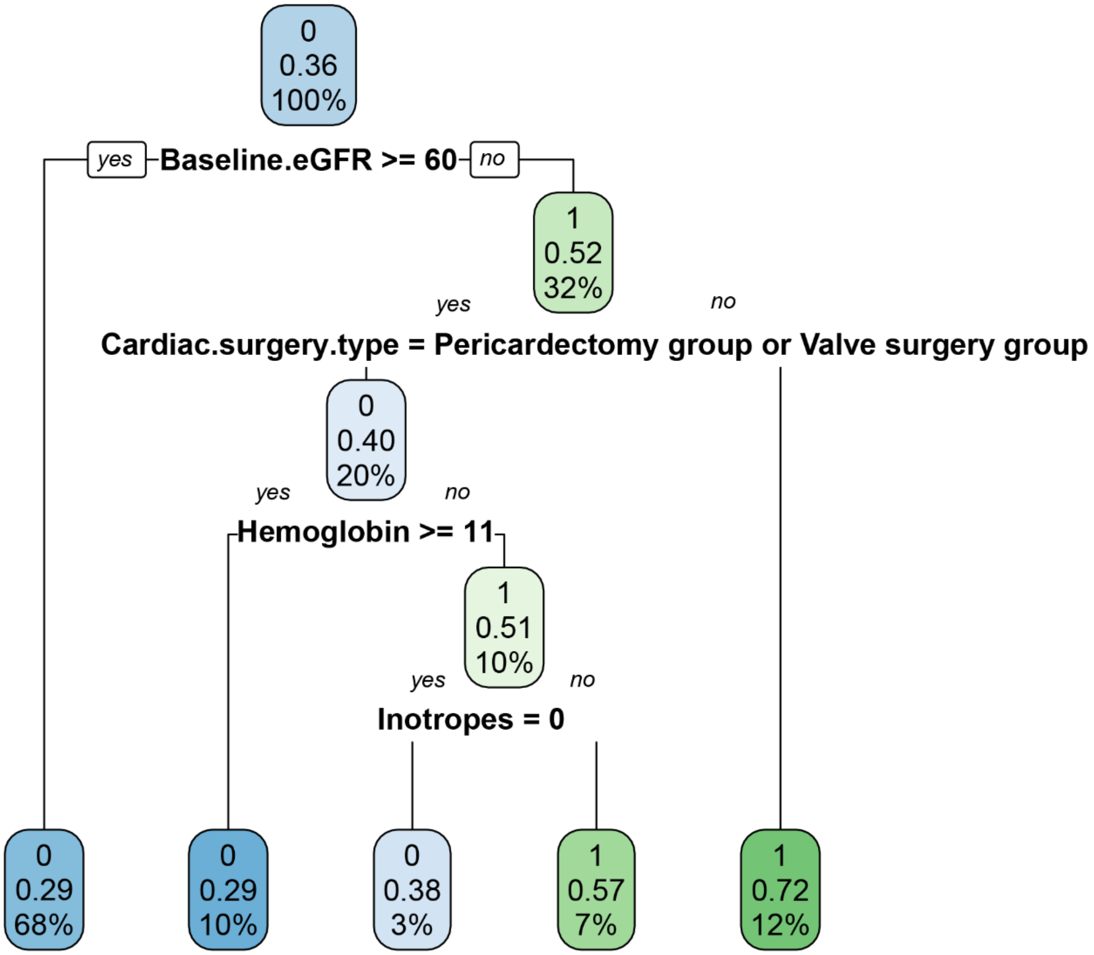

Supplementary Table S1 Leaderboard of top 45 autoML models for CSA-AKI ranked by evaluation metrics using validation dataset

Rank	Model ID	AUC	Log loss	RMSE	MSE
1	StackedEnsemble_AllModels_3_AutoML_1_20211031_170047	0.777477459373283	0.546459347839992	0.428101258420667	0.183270687461359
2	StackedEnsemble_AllModels_2_AutoML_1_20211031_170047	0.773762554202448	0.541472780910445	0.425124160623815	0.180730551946103
3	StackedEnsemble_AllModels_1_AutoML_1_20211031_170047	0.773350035055754	0.541923951699646	0.425351136451643	0.180923589280704
4	StackedEnsemble_BestOfFamily_1_AutoML_1_20211031_170047	0.773241741802089	0.541880114043628	0.425698096353072	0.18121886923863
5	StackedEnsemble_BestOfFamily_3_AutoML_1_20211031_170047	0.772737675781163	0.543015006080206	0.425686048688056	0.18120861204765
6	StackedEnsemble_BestOfFamily_2_AutoML_1_20211031_170047	0.772442939503146	0.542787093883418	0.425595799092307	0.181131784205019
7	GBM_1_AutoML_1_20211031_170047	0.771870771539193	0.545029939918007	0.427050289427436	0.182371949700057
8	GBM_grid_1_AutoML_1_20211031_170047_model_2	0.77171223914723	0.544501614697186	0.426967799142296	0.182301501504416
9	GBM_grid_1_AutoML_1_20211031_170047_model_11	0.770116309187287	0.546966245682808	0.427927798932142	0.183122201098908
10	GBM_grid_1_AutoML_1_20211031_170047_model_16	0.769074126173921	0.545687661410384	0.427632091422274	0.182869205614188
11	GBM_grid_1_AutoML_1_20211031_170047_model_6	0.768387524617178	0.546875946078973	0.427757481549014	0.182976463021155
12	GBM_5_AutoML_1_20211031_170047	0.767743347221664	0.547846265522666	0.428392244515318	0.183519915160872
13	GBM_grid_1_AutoML_1_20211031_170047_model_14	0.765551804366563	0.55048346881313	0.429592621739097	0.184549820652671
14	GBM_grid_1_AutoML_1_20211031_170047_model_7	0.764637452049534	0.551072950563168	0.43104684791713	0.185801385099293
15	GBM_3_AutoML_1_20211031_170047	0.763708027991015	0.549131275569399	0.428857526287251	0.18391877785322
16	GBM_grid_1_AutoML_1_20211031_170047_model_1	0.763258108596921	0.549864223764978	0.429892682456671	0.184807718429792
17	GBM_2_AutoML_1_20211031_170047	0.761695113183196	0.553063273816373	0.431086772286367	0.185835805240278
18	GBM_grid_1_AutoML_1_20211031_170047_model_10	0.75964423991533	0.553470882528734	0.430816680698685	0.185603012368232
19	GBM_grid_1_AutoML_1_20211031_170047_model_9	0.759394718861782	0.554178650562614	0.430772692939089	0.185565112981995
20	GBM_grid_1_AutoML_1_20211031_170047_model_12	0.757099906666845	0.555638148301273	0.431567430420564	0.186250446999809
21	GLM_1_AutoML_1_20211031_170047	0.756497595219913	0.553505293766212	0.431628046457462	0.186302770488685
22	GBM_grid_1_AutoML_1_20211031_170047_model_3	0.753327505593291	0.55841600312415	0.433440402972686	0.187870582929124
23	GBM_grid_1_AutoML_1_20211031_170047_model_8	0.752950153843406	0.560788122592001	0.43500302433334	0.189227631179152
24	GBM_4_AutoML_1_20211031_170047	0.751895690151789	0.560328570623772	0.434198384756095	0.188528237324802
25	GBM_grid_1_AutoML_1_20211031_170047_model_15	0.751718178529802	0.558503796685916	0.433656524835367	0.188057981532287
26	DRF_1_AutoML_1_20211031_170047	0.751522804103086	0.561696310614395	0.434073819875047	0.188420081100914
27	XRT_1_AutoML_1_20211031_170047	0.750703906148824	0.564321529899464	0.435734593358329	0.189864635849148
28	GBM_grid_1_AutoML_1_20211031_170047_model_5	0.750687159769391	0.564779282302757	0.43698478709019	0.190955704148259
29	GBM_grid_1_AutoML_1_20211031_170047_model_4	0.749163797453657	0.563991714533463	0.436664272538075	0.190675686911206
30	DeepLearning_grid_2_AutoML_1_20211031_170047_model_3	0.743165802553488	2.79562336658503	0.784032510934837	0.614706978202786
31	DeepLearning_1_AutoML_1_20211031_170047	0.742032630878537	4.01529192567497	0.603006889452044	0.36361730872663
32	GBM_grid_1_AutoML_1_20211031_170047_model_13	0.737196276498354	0.573362348760472	0.439859917549921	0.193476747067024
33	DeepLearning_grid_2_AutoML_1_20211031_170047_model_2	0.732429698699141	0.799401867531981	0.467196971538785	0.218273010215012
34	DeepLearning_grid_1_AutoML_1_20211031_170047_model_4	0.728321253611636	5.25817090316428	0.58075944210616	0.337281529595458
35	DeepLearning_grid_1_AutoML_1_20211031_170047_model_1	0.721024856092779	4.337114606437	0.597591667897616	0.357115801540654
36	DeepLearning_grid_1_AutoML_1_20211031_170047_model_10	0.71610532802808	0.839130551523699	0.488394471892734	0.238529160175382
37	DeepLearning_grid_1_AutoML_1_20211031_170047_model_3	0.70901658561419	0.668857030587868	0.46202334289069	0.213465569375888
38	DeepLearning_grid_1_AutoML_1_20211031_170047_model_7	0.691003063471011	1.57894249020104	0.52335982625557	0.27390550773826

39	DeepLearning_grid_1_AutoML_1_20211031_170047_model_6	0.6767585931255	1.51597567374466	0.585466655337274	0.342771204511815
40	DeepLearning_grid_2_AutoML_1_20211031_170047_model_1	0.664819541015233	1.20588631138269	0.621203989848743	0.385894397003997
41	DeepLearning_grid_1_AutoML_1_20211031_170047_model_11	0.643044223838806	7.2360007897259	0.727563210285326	0.52934822496069
42	DeepLearning_grid_1_AutoML_1_20211031_170047_model_2	0.639517436330265	1.45472916315543	0.558980899338536	0.312459645825318
43	DeepLearning_grid_1_AutoML_1_20211031_170047_model_5	0.611470600056268	2.16872577289551	0.57796538518834	0.334043986475907
44	DeepLearning_grid_1_AutoML_1_20211031_170047_model_9	0.519775799472154	2.96089524964053	0.600987434021033	0.361185895851186
45	DeepLearning_grid_1_AutoML_1_20211031_170047_model_8	0.506126383809154	6.16567269906814	0.602334403271588	0.36280673336454

Abbreviations: GBM, gradient boosting machine; RMSE, Root Mean Square Error.

Supplementary Table S2. Development of multivariable logistic regression model to predict acute kidney injury after cardiac surgery using stepwise variable selection in the training dataset

	Univariate analysis		Multivariate analysis	
	OR (95% CI)	p-value	OR (95% CI)	p-value
Age (years)	1.02 (1.01-1.02)	<0.001	0.99 (0.99-1.00)	0.01
Male sex	1.22 (1.11-1.33)	<0.001	1.33 (1.19-1.48)	<0.001
Race				
- White	1 (reference)		1 (reference)	
- Black	1.84 (1.26-2.67)	0.10	2.01 (1.29-3.13)	0.002
- Asian	0.75 (0.53-1.06)	0.001	0.95 (0.65-1.40)	0.79
- Other	1.00 (0.76-1.32)	0.98	1.05 (0.77-1.45)	0.74
Body mass index (kg/m2)	1.03 (1.02-1.04)	<0.001	1.01 (1.00-1.02)	0.008
Admission type				
- Elective	1 (reference)			
- Urgent	1.99 (1.74-2.28)	<0.001		
- Emergent	2.02 (1.69-2.41)	<0.001		
Cardiac surgery type				
- CABG	1 (reference)		1 (reference)	
- Valve surgery	0.53 (0.47-0.60)	<0.001	0.51 (0.43-0.59)	<0.001
- CABG + valve surgery	1.41 (1.23-1.61)	<0.001	1.06 (0.90-1.26)	0.46
- Heart transplant	8.16 (4.28-15.55)	<0.001	5.64 (2.78-11.46)	<0.001
- Pericardiectomy	0.61 (0.46-0.82)	0.001	0.78 (0.56-1.10)	0.15
Comorbidity				
- Congestive heart failure	1.38 (1.25-1.52)	<0.001		
- Arrhythmia	1.97 (1.76-2.21)	<0.001	1.47 (1.30-1.67)	<0.001
- Valvular disease	0.98 (0.87-1.11)	0.79		
- Peripheral vascular disease	1.75 (1.61-1.91)	<0.001	1.40 (1.27-1.54)	<0.001
- Hypertension; uncomplicated	1.11 (0.99-1.23)	0.05	1.24 (1.09-1.42)	0.001
- Hypertension; complicated	2.23 (2.04-2.43)	<0.001	1.46 (1.30-1.64)	<0.001
- Paralysis	2.78 (1.95-3.96)	<0.001		
- Neurological disorders	2.13 (1.68-2.71)	<0.001		
- COPD	1.52 (1.37-1.67)	<0.001		
- Diabetes; no complications	1.63 (1.47-1.81)	<0.001		
- Diabetes; complications	1.89 (1.68-2.11)	<0.001		
- Hypothyroidism	1.16 (1.04-1.31)	0.01		
- Liver disease	1.98 (1.64-2.37)	<0.001	1.33 (1.07-1.65)	0.01
- Peptic ulcer disease	2.16 (1.24-3.75)	0.006		
- Lymphoma	1.90 (1.25-2.89)	0.003		
- Solid cancer	1.09 (0.82-1.45)	0.56		
- Connective tissue disease	1.07 (0.88-1.31)	0.48		
- Coagulopathy	2.12 (1.95-2.31)	<0.001	1.56 (1.42-1.72)	<0.001
- Obesity	1.46 (1.33-1.60)	<0.001	1.23 (1.09-1.40)	0.001
	2.37 (1.74-3.23)	<0.001		

- Weight loss	1.98 (1.36-2.88)	<0.001		
- Blood loss anemia	1.62 (1.33-1.97)	<0.001		
- Anemia	1.31 (0.94-1.82)	0.11		
- Drug abuse	3.17 (1.64-6.11)	0.001		
- Psychosis	1.19 (1.05-1.35)	0.006		
- Depression				
Echo finding				
- LVEF	0.97 (0.97-0.98)	<0.001		
- RVSP	1.03 (1.03-1.04)	<0.001	1.01 (1.01-1.02)	<0.001
Systolic blood pressure (mmHg)	1.01 (1.00-1.01)	<0.001	1.01 (1.00-1.01)	<0.001
Diastolic blood pressure (mmHg)	0.99 (0.99-1.00)	<0.001		
IABP use	2.78 (2.04-3.79)	<0.001		
Medications				
- Aspirin	1.31 (1.17-1.47)	<0.001	0.69 (0.59-0.81)	<0.001
- Beta-blockers	1.97 (1.78-2.19)	<0.001	1.25 (1.09-1.44)	0.002
- Digoxin	3.88 (2.64-5.68)	<0.001		
- Anti-anginal medications	1.55 (1.37-1.75)	<0.001		
- Anti-arrhythmic medications	1.36 (1.24-1.48)	<0.001	1.23 (1.11-1.36)	<0.001
- Statins	1.91 (1.70-2.15)	<0.001		
- ACEIs	1.59 (1.32-1.90)	<0.001		
- ARBs	2.05 (1.56-2.70)	<0.001		
- NSAIDs	0.70 (0.59-0.84)	<0.001		
- Benzodiazepine	0.88 (0.80-0.95)	0.003	0.88 (0.79-0.98)	0.02
- Vancomycin	1.06 (0.25-4.44)	0.94		
- Contrast	1.50 (1.26-1.79)	<0.001		
- Diuretics	2.59 (2.28-2.94)	<0.001		
- Calcium channel blockers	1.62 (1.37-1.90)	<0.001		
- Vasopressors/inotropes	1.93 (1.75-2.13)	<0.001	1.36 (1.22-1.53)	<0.001
- Insulin	2.20 (2.01-2.41)	<0.001	1.28 (1.15-1.43)	<0.001
Laboratory data				
- Sodium (mEq/L)	0.99 (0.98-0.99)	0.04	0.97 (0.96-0.99)	<0.001
- Potassium (mEq/L)	0.76 (0.70-0.82)	<0.001		
- Chloride (mEq/L)	0.93 (0.91-0.94)	<0.001		
- Bicarbonate (mEq/L)	0.97 (0.96-0.99)	0.004		
- BUN (mg/dL)	1.05 (1.04-1.05)	<0.001		
- Ionized calcium (mmol/L)	1.75 (1.56-1.96)	<0.001		
- Glucose (mg/dL)	1.01 (1.00-1.01)	<0.001		
- Albumin (g/dL)	0.34 (0.30-0.38)	<0.001	0.74 (0.62-0.87)	<0.001
- pH	5.07 (2.29-11.22)	<0.001		
- pO2 (mmHg)	1.00 (1.00-1.00)	0.54		
- hemoglobin (g/dL)	0.88 (0.86-0.90)	<0.001	0.91 (0.88-0.93)	<0.001
- WBC (10^9 cells/L)	1.05 (1.03-1.07)	<0.001		
- Platelet (10^9 cells/L)	1.00 (1.00-1.00)	0.06		
- INR	2.70 (2.21-3.29)	<0.001		

- Lactate (mmol/L)	1.12 (1.05-1.19)	0.001		
- eGFR (mL/min/1.73 m2)	0.97 (0.97-0.98)	<0.001	0.98 (0.97-0.98)	<0.001
- positive blood culture	1.49 (0.83-2.67)	0.18		

Abbreviations: ACEI, angiotensin-converting enzyme inhibitors; ARBs, Angiotensin II receptor blockers; BUN, blood urea nitrogen; CABG, coronary artery bypass graft surgery; COPD, chronic obstructive pulmonary disease; eGFR, estimated glomerular filtration rate; IABP, intra-aortic balloon pump; INR, international normalized ratio; LVEF, left ventricular ejection fraction; NSAIDs, non-steroidal anti-inflammatory drugs; pH, potential of hydrogen; pO2, partial pressure of oxygen; RVSP, right ventricular systolic pressure; WBC, white blood cell.

Supplementary method

AutoML

```
y <- "AKI"
x <- setdiff(names(train), y)

aml <- h2o.automl(x = x,
                  y= y,
                  training_frame = train,
                  leaderboard_frame = valid,
                  max_runtime_secs = 3600,
                  seed = 123) #default 1 hour 3600 secs, more time, more accurate


# Task Leaderboard Exploration
lb <- aml@leaderboard
print(lb, n = nrow(lb))
best_model <- aml@leader
lb_df<-as.data.frame(lb)
model_ids<-lb_df$model_id
```

Decision Tree

For DT analysis, the number of terminal nodes was determined considering the scree plot showing the relationship between the tree size and coefficient of variance. The decision tree was pruned based on cross-validated error results using the complexity parameter associated with the minimal error.

```
library(rpart)

library(rpart.plot)

classifier = rpart(formula = AKI ~ .,

                   data = training_set, method = "class")

plotcp(classifier)

min_cp = classifier$cptable[which.min(classifier$cptable[,"xerror"]),"CP"]

Rpart_prune = prune(classifier, cp = min_cp)

prp(Rpart_prune)

prp(Rpart_prune, type = 1)

rpart.plot(Rpart_prune)
```

Random Forest

```
library(randomForest)

randomForest(formula = AKI ~ ., data = training_set_scaled, importance = TRUE)
               Type of random forest: classification
                     Number of trees: 500
No. of variables tried at each split: 8
```

XGBoost

library(caret)

```
xgb_trcontrol = trainControl(
  method = "cv",
  number = 5,
  allowParallel = TRUE,
  verboseIter = FALSE,
  returnData = FALSE
)

xgbGrid<-expand.grid(nrounds = c(100,200),
                     max_depth = c(10,15,20,25),
                     colsample_bytree = seq(0.5, 0.9, length.out = 5),
                     eta = c(0.1, 0.2, 0.3),
                     gamma = c(0, 0.1, 0.2, 0.3),
                     min_child_weight = 1,
                     subsample = 1)
set.seed(123)

xgb_model = train(AKI~.,
                  data = training_set_onehot,
                  trControl = xgb_trcontrol,
                  tuneGrid = xgbGrid,
                  method = "xgbTree")
xgb_model$finalModel
```

ANN

Grid Hyper-Parameter Search

```
hyper_params <- list(
  activation=c("Rectifier","Tanh","Maxout","RectifierWithDropout","TanhWithDropout","MaxoutWithDropout"),
  hidden=list(c(20,20),c(50,50),c(30,30,30),c(25,25,25,25)),
  input_dropout_ratio=c(0,0.05),
  l1=seq(0,1e-4,1e-6),
  l2=seq(0,1e-4,1e-6)
)
hyper_params


response = "AKI"
predictors = setdiff(names(train), response)

## Stop once the top 5 models are within 1% of each other (i.e., the windowed average varies less than 1%)
search_criteria = list(strategy = "RandomDiscrete", max_runtime_secs = 360, max_models = 100, seed=1234567, stopping_rounds=5,
stopping_tolerance=1e-2)
dl_random_grid <- h2o.grid(
  algorithm="deeplearning",
  grid_id = "dl_grid_random",
  training_frame=train,
  validation_frame=valid, #we need validation set
  x=predictors,
  y=response,
  epochs=1,
  stopping_metric="logloss",
  stopping_tolerance=1e-2,        ## stop when logloss does not improve by >=1% for 2 scoring events
  stopping_rounds=2,
  score_validation_samples=10000, ## downsample validation set for faster scoring
  score_duty_cycle=0.025,         ## don't score more than 2.5% of the wall time
  max_w2=10,                      ## can help improve stability for Rectifier
  hyper_params = hyper_params,
  search_criteria = search_criteria
)
grid <- h2o.getGrid("dl_grid_random",sort_by="logloss",decreasing=FALSE)
grid
```

```
grid@summary_table[1,]
best_model1 <- h2o.getModel(grid@model_ids[[1]]) ## model with lowest logloss
best_model1
```

Grid ID: dl_grid_random

```
Used hyper parameters:
  -  activation
  -  hidden
  -  input_dropout_ratio
  -  l1
  -  l2
Number of models: 100
Number of failed models: 0

Hyper-Parameter Search Summary: ordered by increasing logloss
        activation           hidden input_dropout_ratio      l1      l2               model_ids logloss
1         Rectifier     [30, 30, 30]             0.00000 0.00003 0.00001 dl_grid_random_model_80 0.56554
2 MaxoutWithDropout [25, 25, 25, 25]             0.05000 0.00006 0.00001 dl_grid_random_model_19 0.56835
3 MaxoutWithDropout [25, 25, 25, 25]             0.00000 0.00001 0.00001 dl_grid_random_model_68 0.56963
4 MaxoutWithDropout         [20, 20]             0.00000 0.00006 0.00002 dl_grid_random_model_84 0.57416
5              Tanh     [30, 30, 30]             0.00000 0.00004 0.00002 dl_grid_random_model_23 0.57763

---
          activation       hidden input_dropout_ratio      l1      l2               model_ids logloss
95          Rectifier     [50, 50]             0.00000 0.00008 0.00002 dl_grid_random_model_96 1.72986
96             Maxout     [20, 20]             0.05000 0.00001 0.00000 dl_grid_random_model_59 1.93356
97          Rectifier [30, 30, 30]             0.05000 0.00006 0.00006 dl_grid_random_model_18 2.01191
98             Maxout     [20, 20]             0.00000 0.00010 0.00009 dl_grid_random_model_99 2.04596
99             Maxout     [20, 20]             0.05000 0.00010 0.00003 dl_grid_random_model_61 2.33901
100 MaxoutWithDropout     [20, 20]             0.05000 0.00001 0.00007 dl_grid_random_model_65 2.41733

ANNBestmodel = h2o.deeplearning(y = 'AKI',
                                training_frame = as.h2o(training_set_scaled),
                                activation = 'RectifierWithDropout',
                                hidden = c(50,50),
                                epochs = 1,
```

```
seed = 1234751,
input_dropout_ratio = 0.05,
l1 = 4.5e-05,
l2 = 3e-06,
max_w2 = 10,
distribution = 'bernoulli',
score_validation_samples = 10000,
score_duty_cycle = 0.025,
stopping_rounds = 2,
stopping_metric = 'logloss',
stopping_tolerance = 0.01,
max_runtime_secs = 350.293,
train_samples_per_iteration = -2)
```

Evaluation indices

In assessing the classification efficiency of all classifiers, a confusion matrix is important. It is a 2-2 matrix that offers details about the real and forecast classifications. There are four components in the confusion matrix: true positive (TP), true negative (TN), FP, and FN. A patient who dies can be classified correctly (TP) or incorrectly (FN), and a patient who survives can be classified correctly (TN) or incorrectly (FP).

The evaluation indices are defined as:

- Accuracy = (TP + TN)/(TP + TN + FP + FN).
- Precision = TP/(TP + FP)
- ERR = (FP+ FN)/(TP+TN+FN+FP)
- MCC = ((TP x TN)-(FP x FN))/sqrt(((TP + FP)(TP + FN)(TN + FP)(TN + FN)))
- F-score = (2 x precision x recall)/(precision + recall).

TRIPOD Checklist: Prediction Model Development

Section/Topic	ı	Checklist Item	Page
**Title and abstract**			
Title	1	Identify the study as developing and/or validating a multivariable prediction model, the target population, and the outcome to be predicted.	1
Abstract	2	Provide a summary of objectives, study design, setting, participants, sample size, predictors, outcome, statistical analysis, results, and conclusions.	2
**Introduction**			
Background and objectives	3a	Explain the medical context (including whether diagnostic or prognostic) and rationale for developing or validating the multivariable prediction model, including references to existing models.	3
	3b	Specify the objectives, including whether the study describes the development or validation of the model or both.	4
**Methods**			
Source of data	4a	Describe the study design or source of data (e.g., randomized trial, cohort, or registry data), separately for the development and validation data sets, if applicable.	5
	4b	Specify the key study dates, including start of accrual; end of accrual; and, if applicable, end of follow-up.	5-6
Participants	5a	Specify key elements of the study setting (e.g., primary care, secondary care, general population) including number and location of centres.	5-6
	5b	Describe eligibility criteria for participants.	5
	5c	Give details of treatments received, if relevant.	5-6
Outcome	6a	Clearly define the outcome that is predicted by the prediction model, including how and when assessed.	5-6
	6b	Report any actions to blind assessment of the outcome to be predicted.	5-6
Predictors	7a	Clearly define all predictors used in developing or validating the multivariable prediction model, including how and when they were measured.	5-6
	7b	Report any actions to blind assessment of predictors for the outcome and other predictors.	7-8
Sample size	8	Explain how the study size was arrived at.	8
Missing data	9	Describe how missing data were handled (e.g., complete-case analysis, single imputation, multiple imputation) with details of any imputation method.	5
Statistical analysis methods	10a	Describe how predictors were handled in the analyses.	5-6
	10b	Specify type of model, all model-building procedures (including any predictor selection), and method for internal validation.	6-8
	10d	Specify all measures used to assess model performance and, if relevant, to compare multiple models.	6-8
Risk groups	11	Provide details on how risk groups were created, if done.	6-8

**Results**			
Participants	13a	Describe the flow of participants through the study, including the number of participants with and without the outcome and, if applicable, a summary of the follow-up time. A diagram may be helpful.	8-9
	13b	Describe the characteristics of the participants (basic demographics, clinical features, available predictors), including the number of participants with missing data for predictors and outcome.	8-9
Model development	14a	Specify the number of participants and outcome events in each analysis.	9-12
	14b	If done, report the unadjusted association between each candidate predictor and outcome.	11-12
Model specification	15a	Present the full prediction model to allow predictions for individuals (i.e., all regression coefficients, and model intercept or baseline survival at a given time point).	13
	15b	Explain how to the use the prediction model.	13-16
Model performance	16	Report performance measures (with CIs) for the prediction model.	13-16
**Discussion**			
Limitations	18	Discuss any limitations of the study (such as nonrepresentative sample, few events per predictor, missing data).	20
Interpretation	19b	Give an overall interpretation of the results, considering objectives, limitations, and results from similar studies, and other relevant evidence.	18-20
Implications	20	Discuss the potential clinical use of the model and implications for future research.	20
**Other information**			
Supplementary information	21	Provide information about the availability of supplementary resources, such as study protocol, Web calculator, and data sets.	Supplementary
Funding	22	Give the source of funding and the role of the funders for the present study.	21
